# Attributable Risk to Assess the Health Impact of Air Pollution: Advances, Controversies, State of the Art and Future Needs

**DOI:** 10.3390/ijerph17124512

**Published:** 2020-06-23

**Authors:** Annunziata Faustini, Marina Davoli

**Affiliations:** Department of Epidemiology, Unit of Etiological and Occupational Epidemiology, Regional Health Service Lazio Region, 00147 Rome, Italy; m.davoli@deplazio.it

**Keywords:** attributable risk, attributable fraction, health impact assessment, air pollution

## Abstract

Despite the increased attention given to the health impact assessment of air pollution and to the strategies to control it in both scientific literature and concrete interventions, the results of the implementations, especially those involving traffic, have not always been satisfactory and there is still disagreement about the most appropriate interventions and the methods to assess their effectiveness. This state-of-the-art article reviews the recent interpretation of the concepts that concern the impact assessment, and compares old and new measurements of attributable risk and attributable fraction. It also summarizes the ongoing discussion about the designs and methods for assessing the air pollution impact with particular attention to improvements due to spatio-temporal analysis and other new approaches, such as studying short term effects in cohorts, and the still discussed methods of predicting the values of attributable risk (AR). Finally, the study presents the more recent analytic perspectives and the methods for directly assessing the effects of not yet implemented interventions on air quality and health, in accordance with the suggestion in the strategic plan 2020−2025 from the Health Effect Institute.

This state-of-the-art review makes a contribution to current knowledge about the impact assessment of air pollution on population health, by reflecting on the relevance of attributable risk to public health and also on the scientific, methodological problems of defining it, with the aim of contributing to future advances of research in this field.

## 1. Aims and Definitions of the Health Impact Assessment and its Measurements 

The impact of exposure on public health is assessed by measuring its contribution to the total disease incidence or mortality [1]. The attributable risk and the attributable fraction are the most important measurements of this impact.

The attributable risk (AR) is the rate (proportion) of a health outcome (disease or death) in exposed individuals, which can be attributed to the exposure [2]. AR assesses how much greater in absolute terms the frequency of an outcome is among the exposed compared with the non-exposed [3]. It is measured as the difference in the rates of an outcome among unexposed individuals (Iu) from the rates among those who have been exposed (Ie), according to the formula: AR = Ie − Iu.

*This estimate is based on the assumption that all other possible causes of the studied outcome have equal effects in exposed and non-exposed individuals.*

The concept of AR was first proposed by Levin in 1953 [4] in the context of studies on cancer, which were mainly cohort studies where people were assembled on exposure and followed over a defined period of time.

*In the light of the discussion about the attributable measures by Greenland and Robins in 1988 [5], Steenland and Armstrong in 2006 [6], Rothman [1] and Greenland in 2015 [7] we underline that this AR is very similar to a difference of incidence proportions or average risks, since it uses as denominators the exposed and not-exposed individuals and not the time-persons.*

The attributable fraction (AF) is the proportion of all cases (or overall incidence) that can be attributed to a specific exposure in a population since it combines relative risk and prevalence of exposure. It is measured as AR divided by the incidence risk in the exposed, according to the formula: AF = ((Ie − Iu)/Ie). Thus giving an estimate of the proportion of cases that would not have occurred if exposure had been totally absent [1,6].

*The no-exposure hypothesis is usually defined as counterfactual—i.e., far from the experience—in a causal approach framework, since it postulates that the same population is followed in an identical situation where only the exposure level changes to the reference value of 0 [8].*

*This estimate as well as AR is based on the assumptions that (1) all other possible causes of the studied outcomes have equal effects in the exposed and nonexposed groups, (2) the studied association is causal.*

*The AF, like the AR, uses proportions or risk fractions, but divides the difference estimated in the AR by the proportion of cases among the exposed, thus giving an indication of the magnitude of excess risk from exposure as a proportion of total incidence, unlike AR.*

To extend this definition to the general population, AR should be theoretically estimated from a life-time follow-up of exposed and non-exposed cohorts in the studied population [6].

In general, using AR, AF and EF similarly in those exposed and in the population as a whole, is not acceptable as it has been pointed out by Rothman [1] and Greenland [7], essentially because populations are not closed cohorts and causality requires many assumptions relative to biological evidence.

Attributable fraction has been renamed excess fraction (EF) by Greenland and Robins [5], to resolve the ambiguity introduced by the term “attributable” whose meaning might be either a case in which an exposure played an etiological role (etiological fraction) or a case that would not have occurred, had the exposure not occurred (excess fraction). The difference is important since all excess cases are etiological cases, but not all etiological cases contribute to the fraction attributable to a specific exposure. The authors then proposed three different meanings of attributable fraction: excess fraction, etiologic fraction and incidence-density fraction, that uses the rate per person-years per year. These definitions should be chosen and estimated according to the questions of the research and to their relevance to public health, as discussed below.

If the essential question is “whether” the outcome occurs by time t due to an exposure, and if then the public health objective is to control the dangerous exposure overall, then the excess fraction is an adequate estimate of an attributable fraction.

If the essential question is “when” the exposure occurs, and the public health objective is identifying the specific age and condition when preventing or reducing exposure, the excess fraction could adequately represent the AF, on the condition that hypotheses about periods or conditions of vulnerability are made explicit.

If the essential question is “whether the exposure caused the outcome”, attributable fraction (AF) by itself is not an appropriate estimate, because according to the traditional definition, the AF measures only the excess cases that could in total be much fewer than the etiological cases, because they are related only to the studied exposure.

We would like to point out here that excess fraction could be an etiological fraction (EF) and therefore, the proportion by which the incidence rate of the outcome in the population would be reduced on condition that the exposure was eliminated; in the above first and second cases, it is also required that (1) a causal (etiological) relationship between the studied exposure and the effect was assessed, and (2) the many biological assumptions about a causal relationship were respected.

AR has been used in the literature to mean various concepts and measures.

These other measures include the population attributable risk (PAR), the population excess rate (PER), and the rate difference (RD), but none of them can be considered entirely equivalent to AF.

Their definitions follow according to the Last dictionary, 2001 [2] to lead to a better understanding in the literature, and to review briefly the possible pitfalls connected to the concept of causality [5,7].

The population attributable risk (PAR) helps determine which exposures are most important in a specific community and is calculated as the incidence of a disease in the total population, minus the incidence in the group of those unexposed to a specific risk factor (It − Iu), thus giving the risk attributable to that risk factor in the population. PAR is often used instead of AF.

It is measured as: PAR = ((It − Iu)/(It × 100), where “It” is the incidence rate for the total population, Iu is the incidence rate among the unexposed, or PAR = (((Pe(Ie − Iu)/(Pt × It) × 100), where Pe is the number of people exposed, Ie is the incidence rate among the exposed, Iu is the incidence rate among the unesposed, Pt is the number of people in the population, and It is the incidence rate in the population.

The population excess rate (PER) is very similar to PAR; it measures the disease associated with exposure to a putative cause in the population, but it is calculated merely as the difference between the rates of disease in the total population and in the nonexposed.

PER = Pt − Pu. The rate difference is used as a measurement that is equivalent to the population excess rate, but it is once again calculated as absolute difference between incidence rate in an exposed population group and a non exposed population group, according to the formula: RD = Ie − Iu.

To complete this brief review of the concepts related to the definition of attributable risk we would also like to consider here the definitions of Health Impact assessment and Accountability.

The definition of Health Impact Assessment (HIA), as published in the Gothenburg Consensus Paper by the WHO Regional Office for Europe in 1999, is “A combination of procedures or methods by which a policy, program or project may be judged as to the effects it may have on the health of a population [9].”

Impact assessment is an important step in public health epidemiology, and can be seen as related to Risk Characterization, the last step in Risk Assessment, as defined by EPA [10], together with Hazard Assessment, Dose–Response Assessment and Exposure Assessment.

Risk Characterization (1) reports the epidemiological results, including estimates of population exposed with harmful effects, (2) makes explicit assumptions and uncertainties, and (3) provides indications for public-health interventions to reduce exposure.

The fields in which impact assessment has been applied include studies about the environment where people live, either natural [11] or man-made (through urban planning or house building [12]), their health care systems [13], their medical treatments [14], but also the clinical prediction models [15] and the impact of reducing exposure through controlling sources and passing legislation to reduce exposure [16].

On the other hand, it has long been recognized that health and its determinants are strongly influenced by policies, programs, and projects outside of the health care sector.

Thus we have the obvious interface between the epidemiological results for the HIA and the more complex Policy Impact Assessments (PIAs), which define formally evidence-based procedures to assess the economic, social, and environmental effects of public policy [17].

These procedures are not analyzed here, nor are the possible interactions with various levels of PIAs. We would like just to touch upon the complexity of PIAs which require many different kinds of expertise. The PIA procedures have been incorporated into policy making in the OECD countries and the European Commission [18].

We would instead like to verify here how much of the prediction made many years ago by IrvaPicciottohas in fact come to pass. She wrote at the time: “A serious appraisal of current methods and practice suggests that much can be done to improve scientific rigor in risk assessment [19].

Accountability which has been termed “evaluating the extent to which air quality regulations improve public health” is part of a broad effort to assess the performance of environmental regulatory policies. The Methods for Accountability Research have been promoted by the Health Effect Institute [20]. One of the strengths of this approach consists in analyzing the compliance to the most important steps of the chain of effectiveness to add or change regulatory actions. In the case of air pollution, the crucial steps involve emissions, ambient air quality, exposure/dose, human-health response.

## 2. Health Impact Assessment of Air Pollution

The health effects of air pollution have been well known to the scientific community since the early 20th century, when serious epidemics of mortality and respiratory diseases were linked to concurrent extraordinary peaks of air pollution in natural experimental or extraordinary scenarios such as London fog [21]; fog in the Meuse valley [22]; Still Mill in Utah Valley [23].

Toward the end of the 20th century, researchers started to focus their attention on the possible adverse health effects induced by “common” levels of outdoor air pollutants [24,25].

Clinicians have also acknowledged the causal role of air pollution on human health since 2000, when the American Thoracic Society (ATS) first defined the concept of an “adverse respiratory effect of air pollution” (ATS, 2000) as “medically significant physiologic or pathologic changes” [26].

All these scientific contributions provided a fundamental support for the definition of standardized legislation on air quality in the United States (U.S. EPA, 1996) and in Europe (European Council, 1999; European Parliament and Council, 2008).

Today’s research is exploring the possible effects of air pollution below the current limits of exposure both for short-term exposure-going from APHENA in Europe and the USA [27] up to the recent almost worldwide studies [28,29]-and for long-term exposure (e.g., in the Netherlands [30] in Europe [31], in the USA [32,33] and in China [34]).

### 2.1. Short-term Effects

In the late 1990s, two important multicity studies-one in Europe (APHEA, 1996) and another in the USA (NMMAPS, 2000)-were started with the aim of providing answers to the unresolved uncertainties which had emerged from previous studies about the short-term effects of air pollution which had been carried out mainly in single cities, apart from the milestone Air Pollution andMortality in Six US Cities [24]. We summarize below the essential points of these studies since they are the basis of many subsequent methodological developments right up to today.

Both APHEA and NMMAPS were multicity studies which benefited from the following advantages over single-city studies:

1. They involve larger populations and can recruit a larger number of cases, thus increasing the power of association;
2. They can observe the same relationship under different circumstances, such as seasons, and in different populations thus making causality a more plausible interpretation of any possible association, providing that heterogeneity has been controlled for or assessed;
3. Even a “weak” effect of air pollution on health will constitute an important public health problem, since a unique characteristic of air pollution exposure is its ubiquity for large populations.

The most important problems with these studies were recognized by the researchers themselves, i.e., (1) not having studied other factors potentially responsible for the effects, including air pollutants other than particulate matter (PM), (2) not having individual measurements of exposure, which could prefigure exposure measurement errors, (3) having measured mortality that was premature only by a few days, which is an effect of limited public-health impact, (4) having used different methods to study this association in different cities.

To respond to the above problems:

1. The researchers thereafter proposed a “Measurement error model for time-series studies of air pollution and mortality” which was a combination of the Berkson model; and the Bayesian hierarchical generalized additive model (GAM).
  The Berkson model [35] deals with the relationship between ambient concentration and personal exposure by comparing the two measurements to estimate the error between them. Although the Berkson error—unlike the misclassification—causes little or no bias in the association, the difference between the average personal exposure and the true ambient level has been identified as an important source of bias in log-linear models [36].
  The Bayesian hierarchical generalized additive model was the tool for modeling variability across the studies of the relationship between personal and ambient exposure concentrations. Two different approaches were available: a hierarchical multivariate regression with missing predictors for either continuous or categorical data [37].
2. They included gaseous pollutants in subsequent studies on air pollution effects. This innovation was supported by using a hierarchical model to assess exposure-health outcome association which made it possible to estimate the independent effects of multiple pollutants in the presence of measurement error [38].
3. They adopted a longer scale of mortality linked to short-term exposure which overcame the mortality displacement [39], and helped to introduce the long-term studies.
4. They took into account the temporal structure of the exposure–response relationship which led to including effects of up to 5 days after exposure peak or harvesting. The distributed lag models played the major role in assessing the new exposure–response relationship of short-term effects [40,41]. Later, the study by Gasparrini and Leone [42] extended the definition of attributable risk within the framework of distributed lag models.
5. They revised the frailty hypothesis—which restricted mortality to the frail people-by showing that larger effects occurred in frail people only at shorter time scales [43].

In parallel, a rich discussion developed about which was the better design, to assess the effects of long-term exposure between time-series and cohort study.

The idea that cohort design might have been more appropriate for assessing effects related to long-term exposure was introduced in NMMAPS in 2000 [44]. The discussion began with Kunzli’s observations [45] about the double action of air pollution on both the long-term underlying diseases and the short term risk of death: the author identified four different combinations: (a) air pollution increases the risks of both underlying diseases, leading to frailty, and of short-term mortality among the frail; (b) air pollution increases the risk of chronic diseases but is unrelated to the timing of death; (c) air pollution is unrelated to chronic diseases but short term exposure increases mortality among the frail; (d) neither underlying chronic disease nor death is related to air pollution exposure. The author concluded that the cohort design was more powerful for studying long-term exposure, as opposed to the time-series approach, because the later captures deaths in categories (a) and (c) only, whereas the cohort design assesses cases in categories a, b and c. Hence the impact assessment of air pollution on mortality should be based on cohort studies.

This position has been discussed claiming that “Lifetime lost due to short air pollution peaks, may not be sufficiently captured in cohort studies because long-term mean concentrations are insensitive to such hidden peaks” [46]. Although no available design could fully assess the contribution of air pollution to life experience, cohort studies might be more powerful for case attribution, if long-term exposure was used, since it also reflects past peak exposures.

The spatio-temporal extension of short-term analysis [47] as well as the studies of the short term effects in cohorts [48,49] contributed important developments in assessing the effects of this sort of cumulative exposure to both short-term and long term exposures:

- The first approach overcame the limited capacity of the time-series so as to take account of spatial variation [47] and the introduction of Cox proportional hazards models in spatio-temporal analysis introduced the possibility of estimating results for different geographic levels, such as cities and states;
- The second approach included in the short-term assessment the effect due to the sensitivity of some population subgroups, which is itself due to long-term exposure [48,49].

The current situation seems to promise further interesting developments in the methods of dealing with spatial-temporal analysis of short-term health effects. Two recent studies show the potential of using the time-series approach in studying numerous areas [28,29], strengthened by the concentration–response function whose important role has been recognized as a metric for impact assessment in addition to the cause-effect metric [50,51,52] (see also below page 12, The role of dose–response curve).

In the above-cited recent multi-area papers [28,29], the contribution of short-term exposure in increasing mortality emerged as independent of long-term exposure, and a no-threshold linear relationship has been confirmed for the PM-mortality function, with continuous increases starting even at concentrations below those of the current guidelines.

It is also worth noting that the authors used the excess fraction to estimate the risk attributable to short-term exposure, thus highlighting the opportunity of regulatory interventions [28,29].

Daily increases of 10 μg of PM_10_ have been associated, within 2 days, with an additional 0.44% (95% CIs, 0.39−0.50) of daily mortality, 0.36% (95% CI, 0.30−0.43) of cardio-vascular mortality and 0.47% (95% CI, 0.35−0.58) of respiratory mortality.

### 2.2. Long-term Effects

The studies of long-term (L-T) effects are characterized by prolonged exposures to air pollution, measured by annual average and by health effects in the exposed population. Health effects are usually measured as all-cause and cause-specific mortality, and as diseases diagnosed in health services (hospitals, emergency rooms, medical offices). Health effects are also estimated as decreased survival which is measured in terms of either life expectancy or years of life lost (YLL). All of these measurements are appropriate for estimating attributable risks or excess fractions, both which are important metrics of risk assessment.

The long-term approach led to important methodological improvements after the first studies on mortality [24,53,54,55], that facilitated estimating ARs:

1. An increasingly accurate assessment of exposure (although not yet at the individual level) ranging from continuous monitoring of PM_10_ and PM_2.5_ components [56] to models for spatial analysis [47] up to the current, almost worldwide coverage [57];
2. A more comprehensive picture of the health effects of air pollution, which include cancer [58,59], metabolic diseases [60], maternal and birth outcomes [61,62], developmental effects [63], cognitive impairment [64] and central nervous system (CNS) diseases [65]. All these additional diseases have a progression that is consistent with cumulative exposure and progressive mechanisms of damage, such as chronic or degenerative diseases;
3. An updated definition of “adverse health effects of air pollution” that includes asymptomatic signs of health deterioration, such as biological effects, altered biomarkers and reduced functions [66];
4. A better comprehension of the mechanisms of damage (systemic inflammation, oxidative stress, immune modulation and epigenetic alterations) [67,68];
5. The extension of exposure-lag-response models to allow for the health effects due to the protracted exposures to environmental factors [69].

The most frequently used approaches for assessing L-T effects were cohort studies and the studies that use functions to link exposure to health effects [11].

Cohort studies had been focused on specific sources that were both noxious to human health and susceptible to reduction, such as traffic density [70,71,72], industrial emissions [73] and home wood combustion [74]. Or they used cohorts created for different reasons but appropriate for representing population and assuring long prospective follow-ups to assess exposure changes after interventions [30,47,75,76,77]. In addition, they also used populations that were resident in defined areas thanks to the availability of wide-ranging administrative and environmental data [78,79,80,81,82].

Studies using functions to link long-term exposure to health effects were promoted by the Global Burden of Disease (GBD) for estimating the burden of diseases attributable to ambient PM_2.5_ in areas with extremely high concentrations of air pollution, but no direct epidemiological evidence [83], such as India [84]. The integrated response function that these studies implemented uses satellite data to assess air pollution and makes deaths data and mortality rates comparable through the use of a standardized method [85]. This makes it possible to compare attributable risks across countries, where, briefly, the risk function to link exposure to health effects is estimated in each country for different exposures (ambient air pollution, second-hand smoke, active smoking and household solid cooking fuel), and the population attributable fractions (PAFs) for each of them are calculated by using worldwide ambient PM_2.5_ concentrations [86].

### 2.3. Health Effects of Abatement in Air Pollution

Both short-term and long-term studies of air pollution effects contribute to health impact assessment, since both of them give estimates of damage due to increasing noxious exposures, by using population attributable fractions or excess fractions, as the metric of effects.

An additional metric called “impact fraction” has also been proposed [87] for assessing “the potential efficacy, effectiveness, adequacy, and efficiency of planned intervention strategies”, in studies that aim to estimate the health gains achievable by potential reductions of air pollutants levels [88,89,90]. This approach could contribute increasingly to supporting the political decision to cut back air pollution, but the methods used in estimating the potential benefits should be further enhanced, so that the HEI indicates it as the first pointer for accountability in the Strategic plan 2020−2025 for understanding the health effects of air pollution, 2nd Mar,2020:1−3 [91].

*The theoretical concept of these potential effect measures is that they are a special case of attributable fraction that compare the effect of an observed exposure with that of a counterfactual case in which exposure is completely absent. As a matter of fact, different potential conditions of exposure are usually chosen [92] (possibly on the basis of the knowledge about the available technologies).*

Hence, a current choice of new limits to which air pollution concentrations should be decreased cannot rely on a counterfactual case that would posit zeroing air pollution, nor on a safe tested level, as we know that no threshold exists in the dose–response curve of air-pollution and health effects. Finally, the definition of an “acceptable” damage not based on scientific evidences should involve larger social, economic and-political institutions.

These studies have been and could be still useful also in revising the methods used to predict the possible beneficial effects of interventions, along the same lines as what the HEI did by supporting, in the 2000s, intervention studies on air pollution to test whether the proposed methods were able (1) to assess the population exposure, (2) to identify an effective dose of exposure (3) to identify the best health outcomes (defined as those associated with several pollutants and detectable shortly after exposure change), (4) to identify the best populations as those which make it possible to assess differences between areas, and to study the effects related to intervention [93].

Other studies help in choosing priorities for the intervention studies on the basis of the observed risk assessments for different exposures (behavioral, environmental, occupational), like those provided by the GBD studies [94], or from different sources (industrial, residential, vehicular and multiple sources) with respect to their effects on mortality, morbidity and on changes in pollutant concentrations [95].

## 3. When Scientific Evidence is clear enough to Promote Interventions

### 3.1. The Role of the Dose–Response Curve in Assessing a Causal Relationship between Air Pollution and Health Effects

Promoting an intervention to reduce air pollution with the purpose of reducing the health effects, requires evidence of a causal relationship between exposure to that pollution and those effects. It also requires estimates of excess fractions for the exposure so as to be able to evaluate to what extent the interventions have met expectations.

A no-threshold linear concentration–response (C-R) curve has been assumed to exist by researchers on the basis of a linear relationship of the observed intermediate values in most of the initial studies on air pollution effects. Likewise, a further assumption was that a linear relationship of not measured values there could be on both the left curve, i.e., at the lowest levels of pollutants, and the right one, at the highest levels.

The assumptions about the lowest levels of pollutants were discussed from the point of view of public health, since the shape of the C-R curve is an important parameter for predicting the benefits of reducing high-level exposure [50,51]. Just a bit of evidence of a threshold was found, whereas the two methods of assessing regression linearity (penalized splines and model averaging) were equivalent.

The possibility of predicting C-R curves at the highest levels at which to intervene first of all so as to reduce damages, has been discussed most recently by Cox [96]. The author questions the possibility that the observed C-R relationship might be used to predict the effects of an intervention at the highest levels of PM_2.5_, because this would imply that the magnitude or the direction of the observed curve was similar to those of the predicted curve after intervention. This assumption could not be necessarily true, since the observed values are fixed, whereas the predicted values are based on changing pollutant levels. Additionally, Pope, even though he recognized that both the burden of disease attributable to PM_2.5_ and the benefits of reducing pollution depend upon the observed C-R function, found that pollution abatement may yield greater benefits in relatively clean areas than in highly polluted ones. Hence, he concluded that the shape of the C-R function should be further explored to better understand this relationship for more effective abatement of air pollutant effects in the future [97].

### 3.2. The Adoption of New Analytic Perspectives and Statistical Methods to DirectlyAssess the Effects of Air Pollution Interventions on Air Quality and Health

New types of evidence were proposed for estimating air pollutant regulatory strategies on the basis of direct experiments, rather than deriving predictions from observed situations [98]. The most important feature of this approach-that justifies the use of the term ‘causal inference’-is that different areas are analyzed to allow for the assumption that the intervention to reduce exposure in certain areas is assessed at random, as in a hypothetical randomized experiment. Thus the researchers “anchored” the exposure assessment to the estimates of the causal consequences of well-defined interventions, and could conclude that the observed health effects, observed after the intervention, had diminished due to a given reduction in concentrations.

The new methods were applied by Zigler and colleagues to two case studies:

- The first one, the “causal” health impact on mortality-among Medicare beneficiaries-in areas which exceeded the PM_10_ limits, was compared with the mortality rates among Medicare beneficiaries in areas where PM_10_ limits were not reached. The members of the first group were assumed to be randomly assigned to “treatment”, after testing their comparability with the second group (controls) for potential confounders by using the propensity-score method. In other words, this causal approach allows the consideration of mortality rates among controls as the mortality that might have occurred among cases had their area’s exposure been below the PM_10_ limit.
- The second case study examined the extent to which sulfur dioxide (SO_2_) affects emissions of SO_2_, nitrogen oxides (NO(x)), and carbon dioxide (CO_2_). The authors tested a range of scrubber technologies to reduce multiple gaseous pollutants (SO_2_, NO_2_ and CO_2_) in emissions and outdoor PM_2.5_ concentrations in areas where plants were either equipped with scrubbers or were not so equipped. The causal estimates were supported by applying principal stratification and causal mediation methods to assess the exposure, while a Bayesian nonparametric method was used to evaluate whether the effect on PM_2.5_ was really mediated by reducing gas emissions.

Scrubber systems (chemical or gas scrubbers) control air pollution by removing particulate matter and/or gases from industrial exhaust streams.

Propensity scores [99], principal stratification [100], causal mediation analysis [101], spatial hierarchical models [102], and Bayesian estimation [103] are the methods that support and allow us to evaluate a causal relationship [98].

By grounding accountability research in a potential-outcome framework and applying these methods to national data sets, the authors provide additional evidence of the health effects of long-term, large-scale air quality regulations.

### 3.3. The Question of When the Scientific Evidence is Clear Enough, is still an Ongoing Discussion

The experiences briefly presented above remind us of the difficulties of this discussion. If, from a scientific point of view, it is always possible and appropriate to improve on the available knowledge, as was done in developing the first studies on air pollution effects (see above pp. 5–7), from a public heath point of view, taking a decision on the basis of the contemporaneously available knowledge (as it was done at the end of the 20th century in both Europe and the USA) would be at times desirable or even necessary.

Epidemiologists have expressed doubts about policy indecision in the face of clear scientific evidences [104,105].

On the other hand, the problems of uncertainty [92], verification of hypothesis [89,90] and heterogeneity still affect important studies. Uncertainty is due to errors in estimating mean values in a population, especially when parts of it are commuters; uncertainty may have multiple explanatory factors and is currently quantified by Montecarlo simulation, a statistical technique requiring many assumptions. Interventions implemented to reduce traffic have not achieved the expected results, but the verification of this hypothesis could rely on many factors not considered in carrying out the interventions such as the spatial and temporal variability of a similar “preventive” intervention. Heterogeneity, the first recognized in the multicity studies, has been partly explained by the different composition of particulate matter in different areas [106], or controlled by using hierarchical Bayesian models to combine the city-specific estimates [107] but the discussion about the assumptions of these models is still open [108,109].

We can but wish for increasingly well-defined research questions, more accountability studies and further improvements in research methods so as to provide more solid answers to the scientific questions and the policy indecision. Meanwhile we would like to go back here to what Pope and Dockery wrote in 2006 about the effects of fine particulate air pollution [53]. “Despite important gaps in scientific knowledge and continued reasons for some skepticism, a comprehensive evaluation of the research findings provides persuasive evidence that exposure to fine particulate air pollution has adverse effects on cardiopulmonary health”.

## 4. Health Impact Assessment of Air Pollution to Evaluate Interventions of Exposure Reduction and the Effectiveness of Legislation

The accountability studies, as we saw before, arose as an attempt to “evaluate the extent to which air quality regulations improve public health to assess the performance of all environmental regulatory policies” [20]. We return now to this first mission of accountability, to reconsider its strengths and weaknesses.

Three fundamental steps were identified in evaluating these regulatory policies, in logical sequence they are as follows: (1) reducing emissions, (2) improving ambient air quality, (3) reducing adverse health effects; we could summarize these steps as the impact of air quality improvement on public health.

A few of the most important accountability studies which applied these criteria are reported as follows:

1. The ban of coal sales in Dublin was followed by an important decrease in black smoke concentrations (70%); natural mortality decreased by 5.7%, respiratory mortality by 15.5% and cardiovascular mortality by 10.3% [110].
2. Reducing the sulfur content in fuel in Hong Kong was followed by a substantial reduction in seasonal deaths during the first 12 months, followed by a peak death rate in the subsequent cool-season. It seemed that the intervention led to a significant decline in the annual trend of deaths from all causes (2.1%; *p* = 0.001), respiratory (3.9%; *p* = 0.0014) and cardiovascular (2.0%; *p* = 0.0214) diseases, but not from other causes. The average gain in life expectancy attendant upon the lower pollutant concentration was 20 to 41 days [111].
3. Some measures to reduce traffic were implemented in more than one situation. The results in these cases are conclusive as regards neither exposures nor health effects.

Among the initial studies, those carried out in Atlanta during the Olympic games have produced contradictory results. The first [112] found promising evidence, as the decreased led to a prolonged reduction in ozone levels and lower rates of childhood asthma events. The other study [113] instead found that meteorological conditions contributed significantly to the reduction in ozone, emergency department visits did not decrease, and the strategy adopted for the occasion would not be sustainable beyond that exceptional event.

In contrast, both the studies carried out in London to assess the impact of the congestion charging scheme (CCS) did not find conclusive results:

- The first, appearing five years after the introduction of the CCS, showed a modest benefit in air pollution levels and life expectancy. The explanation given was that the greater reductions in air pollution in deprived areas have had only a small impact in counteracting the socioeconomic inequalities in exposure and mortality rates [114].
- The second study, published eight years after the introduction of the CCS, deals with the oxidative potential (OP) of PM as a parameter of traffic exposure. It shows a remarkable variation of OP between roadside and urban background locations, which was attributed to varying PM components. This result is consistent with the increased vehicle use throughout London in recent years and a decreased number of vehicles entering the CCS [115].

Another well-conducted study evaluates, in several Dutch cities, the air quality and health effects of local traffic policies including low emission zones; however, apart from one urban street in The Hague where traffic flow and air pollution were drastically reduced, the study did not find that “reductions in air pollution related to abatement policies lead to actual improvements in respiratory function” [116].

## 5. Conclusions

The experience that the world is currently facing thanks to Covid-19 reduces mobility-worldwide and simultaneously-as regards both long distance and local urban traffic, thus offering the framework for a “natural” experiment which the Health Effect Institute also suggested to study [117] more specifically about the health effects of reducing traffic and long-distance transports. What has emerged until now are few points which, however, support the hypothesis of a natural experiment, such as the reduction of particulate matter in the atmosphere during the pandemic [118], the association of air pollutants (PM, NO_2_ and O_3_) with an increased risk of Covid-19 infection [119] and a possible role in transmission of severe Covid-19 infections by air pollutants [120].

Some people would like causal accountability studies to support the more effective political interventions and thus replace traditional assessment which would be based on expensive studies about the exposure–response relationship in estimating the burden of diseases [93]. But the authors themselves of the causal accountability studies have concluded that their methods are “an important addition to the toolkit and should continue to be further explored, but cannot wholly substitute for accountability assessments that rely on evidence from other scientific methods, including more traditional epidemiology analyses.”

In general, the impact of the interventions on public health should be maintained both to verify the full achievement of the results and to identify the potential necessary corrections, since the process of accountability involves so many scientific, administrative and political institutions.

In addition, it is possible that many interventions together, or in sequence, are needed to reduce pollutants or that they require more time to exert effects on total population health and so on. This is well evidenced in a not very recent paper which anticipated the importance of longer term, wide-ranging actions or events such as the complex changes associated with the reunification of Germany [121]. This approach is also evocative of experiments that respect the natural times for changes.

Both the kind of accountability that assesses the regulations already in place, and the causal accountability approach, which promises stronger support to the future air quality scenarios, might be necessary for a long time.

## Note

Manuscript has neither been published previously nor is under consideration for publication in another journal.
